# Potential for greenhouse gas (GHG) emissions savings from replacing short motorcycle trips with active travel modes in Vietnam

**DOI:** 10.1007/s11116-023-10394-0

**Published:** 2023-05-08

**Authors:** Yen Dan Tong, Tek Maraseni, Phuong-Duy Nguyen, Duc-Anh An-Vo, Julio Mancuso Tradenta, Thuy Ai Dong Tran

**Affiliations:** 1 School of Economics, Can Tho University, Can Tho, 94000 Vietnam; 2 Centre for Sustainable Agricultural Systems, Institute for Life Sciences and the Environment, University of Southern Queensland, Toowoomba, QLD 4350 Australia; 3 Centre for Applied Climate Sciences, Institute for Life Sciences and the Environment, University of Southern Queensland, Toowoomba, QLD 4350 Australia; 4 La Trobe Business School, La Trobe University, Melbourne, VIC 3086 Australia; 5 Can Tho Institute for Socio-Economic Development Studies, Can Tho, 900000 Vietnam

**Keywords:** GHG emissions, Human mobility, Travel pattern, Travel behaviour, Active travel

## Abstract

In reducing greenhouse gas (GHG) emissions, there is a recognition triggered by the pandemic of the role that walking and cycling (active travel) can make to substitute motorized travel, particularly on short trips. However, there is a lack of evidence at the micro level on the realistic, empirically derived, potential of these options. Here, we used reliable tracing data to examine the potential of these mitigation options for reducing GHG emissions in Vietnam. Apart from similar categories of travel purposes as in other studies, we decided to categorize “visit relatives” and “eating out” as two more separate categories of travel purposes in Vietnamese case, which together accounts for nearly 16% of total trips. We discovered that 65% of all motorcycle trips in this case study were less than 3 miles in duration, therefore active travel was able to create a significant impact on GHG emissions from personal travel. Active travel can replace 62% of short motorcycle trips if considering travel patterns and constraints while saving 18% of GHG emissions that would have come from motorized transport. If active travel can further replace all shopping trips normally done by motorcycles, in total being equivalent to 84% of short trips, 22% of GHG emissions from motorcycles can be reduced. It should be noticed that active travels have time cost implications, impacting economy at both household and city levels, but from a comprehensive “co-benefit” standpoint, this transformation could act as a catalyst for addressing traffic congestion, air pollution, and even community health and well-being in urban areas.

## Introduction

The transport sector is one of the main and most rapidly growing generators of greenhouse gas (GHG) emissions worldwide. According to the International Energy Agency (McBain and Teter 2021), the transport sector generated approximately 8.5 GtCO_2_ in 2019, and 7.2 GtCO_2_ in 2020 regardless of the pandemic’s impacts. It accounts for about 37% of global GHG emissions from end-use sectors (IEA 2021). Amongst all types of transportation modes, road transport is the largest contributor to transport sector emissions and is mainly responsible for their growth in the last five decades. In Energy Technology Perspectives report (IEA 2020), the global transport demand is projected to double, and car ownership rates to increase by 60% by 2070. These factors would result in a large increase in transport emissions. Hence, it is not surprising that the transport sector and road transport in particular, remain key components demanding studied strategies to mitigate GHG emissions worldwide (Schiller and Kenworthy 2017).

Cities around the world have faced difficulties in getting a right movements and necessary information to properly plan their low GHG emissions public transport systems (Harris et al. 2020; Ku et al. 2021; Li et al. 2021; Song et al. 2018). These cities, especially cities in developing countries, are seeking solutions suitable for their features of large population and unsynchronized urban infrastructure (Dong et al. 2018; Zhang et al. 2019). For example, they may consider the improvements in urban infrastructure and material use (Huang et al. 2019), the replacement of transportation technologies (Yang et al. 2020), or the provision of the right economic incentives to encourage people changing their behaviors (Neves and Brand 2019; Nieuwenhuijsen 2020). The selection and successful implementation of any of these policies is challenging, for example they can be costly to implement. Facing potential large costs, policymakers need accurate information on the mitigation benefits when choosing the right policy option (Tong et al. 2020). Thus, the main challenge regarding these policies relates to the accurate estimation of their mitigation benefits which normally requires reliable micro-level empirical evidence on the effect of behavioral changes on total emissions. However, there is a lack of such evidence, which makes the identification of the right policies a very difficult task (Neves and Brand 2019).

Our focus in this study is on-road transport emissions. We consider emissions produced by individuals when traveling within urban areas using motorized transportation, and travel patterns of people there. To our knowledge, this study is the first micro-level evidence in developing countries, which assesses individual mobility behaviors and their associated GHG emissions and mitigation potential. It should be noted that there is little or no data-based evidence so far on the options for reducing transportation-induced GHG emissions in Vietnam, and developing countries (Asian Development Bank 2017; The World Bank Group Initiative 2021). Providing the potential benefits of soft options on emissions reduction will foster the development of urban policies towards more sustainability, especially in rapidly urbanized areas like Vietnam.

Microdata on distance, frequency and the purpose of urban trips are particularly scarce in developing countries. Data with similar reliable in-depth information are resource-intensive and have, to the best of our knowledge, only been produced in developed countries using GPS technology. For example, Neves and Brand (2019) used such technology to estimate a 5% GHG mitigation potential from substituting short motorized trips with active travel modes in Cardiff, Wales. The data that we use in our study offer an unprecedented opportunity to conduct a comparative analysis in Vietnam. This is important for the following two reasons. Firstly, transport demand per capita in developing countries is expected to grow at a rate faster than in high-income countries in the next decades (IEA 2020), with a resulting rapid increase in GHG emissions. Secondly, although studies conducted in developed countries may offer some valuable information for developing countries, the behaviors they study may differ significantly across countries with different levels of development.

This paper makes several contributions to the current literature. Firstly, with some exceptions (e.g. Dissanayake and Morikawa 2008; Jaff and Hamsa 2018; Subbarao and Rao 2014), little research has been carried out on the micro travel behavior data (i.e. people’s travel diary/history) in developing countries due to the costly nature of the tracing method. Our study is the first attempt to characterize people’s travel behavior in Vietnam. The study will provide a useful addition to the relatively small amount of research that has examined people’s travel behavior in the developing world. Secondly, the study is highlighted with the feasibility analyses of potential options for reducing GHG emissions, i.e. substituting short trips by walking or cycling. Previous works on these options were completed in both developed and developing country settings (e.g. Neves and Brand 2019; Subbarao and Rao 2014), however, it will be relatively different in the context of a country where the majority of people use motorbikes as means of transport like Vietnam. The results of this study hence provide a better understanding of the importance of active travel modes in Vietnam.

## Data and estimation method

### Case study: Da Nang, Vietnam

Da Nang, with a population of 1,134,310 people in 2019, is Vietnam’s fifth largest city in terms of population (Da Nang Department of Statistic 2019). It is divided into eight administrative districts, including 06 urban districts, 01 suburban district, and 01 island district. Overall, the city covers an area of over 1,285 km^2^ that connects the north and south axes of Vietnam by road, rail, sea, and air. It is also part of the East–West Economic Corridor connecting Vietnam with three other inland ASEAN countries (Laos, Myanmar, and Thailand). These geographical advantages have contributed to the transformation of Da Nang into an important socio-economic hub in central Vietnam.

**Fig. 1:**
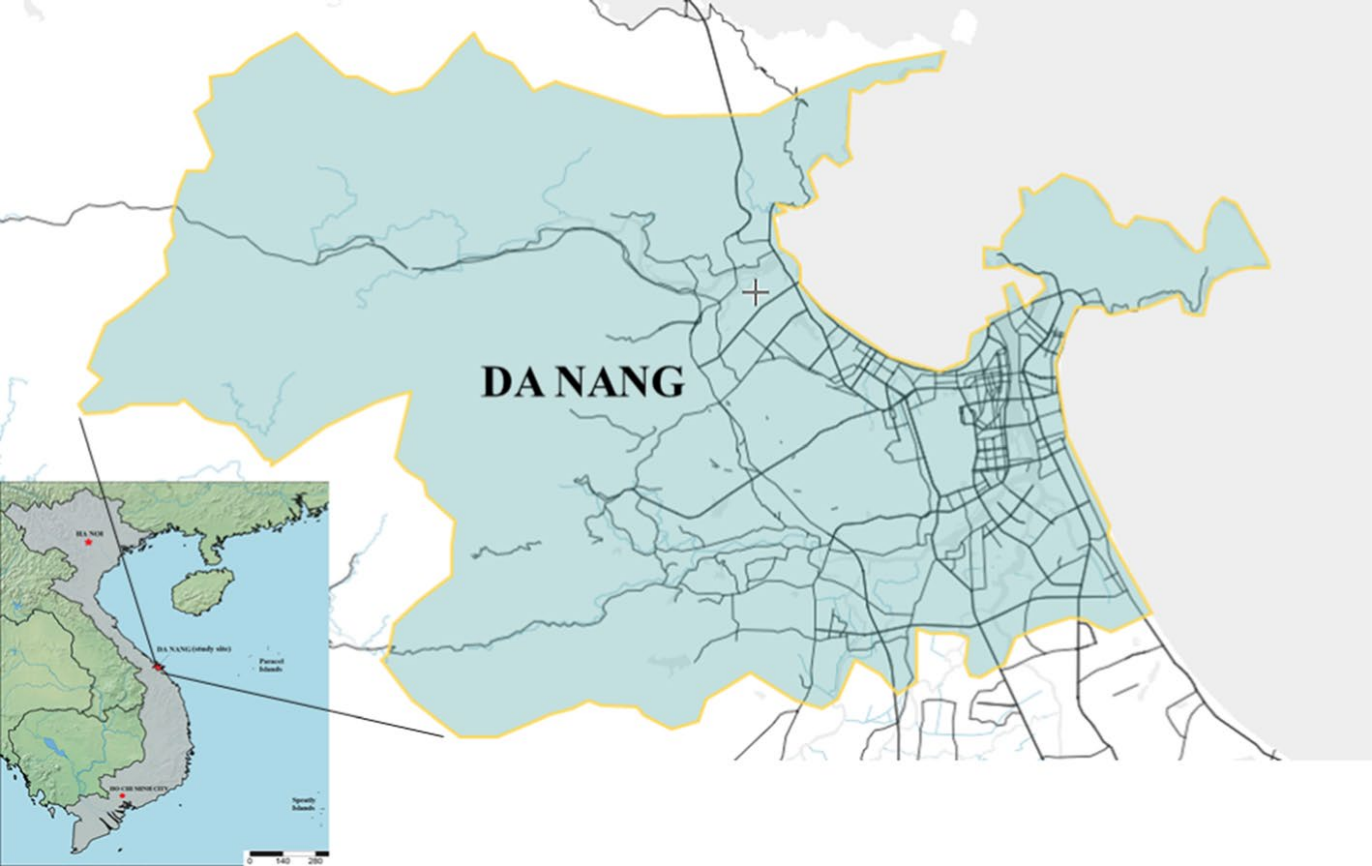
Location of study site – Da Nang, Vietnam

As in any city of the dimensions of Da Nang, motorized transportation constitutes a large fraction of total urban mobility. Motor vehicles comprise about 78% of road traffic (Kutani et al. 2015), and, similarly to Ha Noi and HCMC, motorcycles are the overwhelmingly dominant mode of transportation, comprising more than 80% of the total number of trips (Chu et al. 2019). In terms of the ownership of private vehicles, we do not have Da Nang’s data. However, the figure for Vietnam, in general, is constantly increasing overtime at a strong rate. As of 2020, there were 45 million motorcycles and 3.5 million cars in Vietnam, excluding un-registered vehicles (MONRE 2020). As a consequence, toxic emissions from private vehicles exacerbated the deterioration of the environmental quality, especially air quality in Vietnam. The Air Quality Index of Vietnam is identified as relatively poor. This index was computed by combining three indicators: PM_2.5_ exposure (fine air particulates smaller than 2.5 µm), household solid fuels, and ground-level ozone exposure. In particular, Vietnam’s air quality index was estimated at 32.00/100 points, ranking 115 amongst 180 countries calculated (Wendling et al. 2020). The level of air pollution indicated that much more effort will be required to fight its effects, and to protect human well-being Fig. 1.

### Data collection

The micro-level contact tracing data that we used in our study was collected by epidemiological investigation officers and made publicly available on the NCOVI^1^ dashboard. The dashboard is a public online platform established by the Vietnamese Ministry of Health for the management and publication of information generated during the pandemic. It is an important tool that provides information transparency and real-time updates on the COVID-19 infection and transmission developments. The data includes gender, age, home address, type of job, workplace address, personal contacts, travel history, and purpose of travel of those who tested positive for COVID-19.

This unprecedented dataset does not have the self-reporting bias issue as usually experienced by other academic surveys which are conducted in a normal setting due to the collection of complete and accurate information was a top priority in Vietnam during epidemiological contact-tracing investigation (Pollack et al. 2021). The success of investigation is explained by the country’s culture of surveillance. People are expected to inform authorities about their neighbors’ actions if they suspect any wrongdoing, like disclosing false information or escaping from social isolation (Fleming 2020). The use of private and public security camera systems and the mobile contact tracing application (Bluezone) have also been identified as key in contact tracing success. They were used by the epidemiological teams to track the locations that patients have visited and people they have closely contacted. The different sources used in contact tracing allowed authorities to cross-check the information collected.

In total, the data extracted from the dashboard in August 2020 was constituted from 100 out of 172 observations (patients) that were displayed in the public dashboard during the outbreak in Danang after we excluded observations that potentially may cause bias in data. We used two main criteria to eliminate potentially problematic observations. First, we excluded inpatients in hospitals because the second outbreak in Vietnam started at one of the biggest hospitals in Da Nang. Hence, the data set contained a large number of observations of inpatients. However, these observations were problematic because hospital patients are restricted in their movements, which significantly reduces the variability of their travel schedules. Second, we discarded those who were travelers. As mentioned above, Da Nang is a popular tourist destination. Before the second COVID-19 outbreak the country had entered a stage of relative normality, which allowed individuals to travel domestically without restrictions. Therefore, it could be conjectured that the second Da Nang outbreak included visitors who contracted the disease. Since the purpose of this study is to investigate regular travel behavior, these observations were removed.

Another consideration is that this sample, ideally, should have increased in size till the time we got enough data to analyse, its size instead was determined by the contact-tracing situation. Nevertheless, in relation to the information needed and the sample size, 100 observations is a reasonable number for analysis. We believe the readily free and highly reliable in-depth data utilized in this study is worth a trade-off with this size limit. In comparison, to be able to obtain such detailed data as in our sample, a previous study of Neves and Brand (2019) had to employ a combination of GPS logger, 7-day travel history, and interviews. As a result, only 50 respondents agreed to attend such a complex data collection process in that study. A similar phenomenon has also happened in other studies such as 191 respondents in Stewart et al. (2017) and 39 respondents in Marra et al. (2019).

### Indicators and greenhouse gas emissions calculation

Our analysis was conducted using travel-tracking data collected from 100 individuals in Da Nang, Vietnam. The data present an exceptional opportunity to study urban mobility behaviors and associated emissions and mitigation potential being valuable to policymakers because they include detailed individual information on the trip frequency and distance travelled, as well as the purpose of each trip.

To classify the trips observed in this study, we followed an approach similar to the method proposed in previous studies (e.g. Brand et al. 2013; Neves and Brand 2019). In their research, there divided trip purpose into 4 categories (business trips, travel to work or education, social and leisure, shopping and personal business). We however classified all trips by journey purpose into 6 categories: (1) working or studying, (2) shopping and doing groceries, (3) social activities and leisure, (4) personal business, (5) eating out, and (6) visiting relatives. For the Vietnam case study, we perceived that “visiting relatives” is a significant group that should be treated as a separate category. Vietnam’s family structure is not the same as that of Western countries. Most families in Vietnam today are identified as nuclear in structure, however, they still maintain a close relationship with relatives as a kinship system (Belanger 2000). Hence, movement to visit and look after each other is made regularly amongst kinsmen. As a result, we did not include “visit relatives” in a common category of “social and leisure” as Brand et al. (2013) and Neves & Brand (2019) did. In addition, by observing the habits of Vietnamese people, they tend to have breakfast and lunch in food vendor instead of at home. For that reason, we decided to treat “Eating out” as a separate category of travel purpose. In terms of personal business, these were mainly activities related to medical treatment and examination, administrative procedures, service use, and religion-related activities.

Indicators computed in this study consisted of average distance traveled (by the trip, tour, day, and purpose), trip rate, tour rate, mitigation option. These concepts will be further explained in the Results section.

There were two methods for calculating GHG emissions from transport sources: the fuel-based method and the distance-based method. For the fuel-based calculation, emissions were computed using the multiplication of the three indices, including the fuel used data, heating value, and its emission coefficient. In our study, however, this method was inapplicable as we lacked details on fuel economy data, and the unavailability of fuel type and fuel consumption. In this case, the distance-based method was adopted to calculate GHG emissions.

**Fig. 2:**
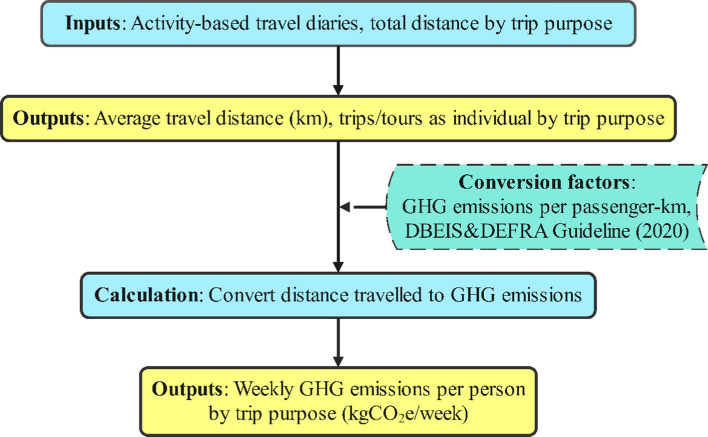
GHG emissions computation method for motorized modes in this study

As shown in Fig. 2, calculating GHG emissions required two indicators: average travel distance, and conversion factors. Average travel distance was computed from our data sample, while conversion factors applied in this study were based on the conjoint guideline between Department for Business Energy & Industrial Strategy (DBEIS) and Department for Environment Food & Rural Affairs (DEFRA). The guideline on methodology was produced by Hill et al. (2020) on behalf of DBEIS & DEFRA. For non-CO_2_ greenhouse gases (methane CH_4_, nitrous oxide N_2_O), the conversion factors were consistently presented as CO_2_ equivalents (CO_2_e). This converting method was employed in previous studies and analyses (Brand et al. 2013; Neves and Brand 2019).

Due to the nature of our available data, we are unable to take into account motorcyle technology details, trip speeds and excess emissions from ‘cold starts’, and instead, we applied a more general GHG emissions calculation, namely GHG emissions conversion factors. It may result in a less accurate assessment of GHG emissions. It worths noting that the higher ambient temperature, the less significant cold-start emissions are (Bielaczyc et al. 2011). Consequently, cold-start emissions are expected to be less significant in such a tropical climate country as in Vietnam.

Conversion factors for motorized sources were divided into three categories based on cylinder capacity: small (mopeds/scooters up to 125 cc), medium (125-500 cc), large (over 500 cc) motorbike. In the case of Vietnam, most mopeds and scooters we observed on the road had a cylinder capacity ranging from 100 to 125 cc. Given no further available data on cylinder capacity in our dataset, choosing the conversion factors of the smallest category for calculation ensured that our analysis was conducted conservatively. Microsoft Excel v2016 and STATA v10 were used to perform data entry, descriptive analysis, and computation of emissions.

## Results and discussions

### Sample description

The sample are evenly distributed between the urban areas and suburbs by population size. This is a sign of a good sample representation of the total distribution of the population in the city. In particular, concerning home address, 39% of the respondents lived in the central business districts (CBD) of Da Nang, which is formed by the areas of Hai Chau and Thanh Khe. According to the Da Nang Population and Housing Census 2019, the share of the population living in Hai Chau and Thanh Khe was around 34% of the total population of Da Nang (Da Nang Department of Statistic 2019).

These sample sizes however are likely biased on selected characteristics, which may cause the limited implications. As shown in Table 1, The sample has an unequal distribution in terms of gender (66% female, 34% male) and marital status (65% married). The age range extends from 13 to 71 years old, with an average age of about 40 years old (± 13.66). Table 1 also shows the distribution of subjects by career, where we observed a larger number of participants grouped around skilled jobs such as healthcare (17%), desk worker (14%) and education (11%).

**Table 1:** Sample distribution based on selected characteristics (N = 100)

Category		Percentage (%)
Gender	Female Male	66 34
Marital status	Married Single	65 35
Job	Student Desk worker Health worker Factory worker Teacher Civil servant Shop-owner Service Retired people or homemaker Missing value	7 14 17 8 11 5 6 5 2 25
Home address	Central business districts (CBDs) Otherwise	39 61
Number of days recorded	From 1 to 3 days From 4 to 7 days From 7 to 14 days Over 14 days	16 54 28 2

*Source: Authors’ calculation*

Based on the travel recorded profile (by days) of respondents, we found that more than half of them had from 4 to 7 recorded days,^2^ and 84% of them had at least 4 recorded days and up to 14 days. The number of recorded days as mentioned in our profile was in line with the recommendations for the needed recorded days from other previous studies on a similar research topic. For instance, the use of multi-day travel history, ideally over the four days, was recommended by Axhausen et al. (2007), to record richer travel data; or the approach of a 7-day-travel diary proposed by Neves and Brand (2019).

### Overall travel activity

First of all, we provided the general concepts on trips and tours (round trips). Trips were identified based on the notion of one-way movement between two activity nodes. For example, the two activity nodes were illustrated as an A node and a B node (all called locations). An individual moved from an A node to a B node in a specified distance that was assigned as a trip. Meanwhile, tours (round trips) were defined based on the notion of all travel activities between A and B. This meant that an individual moved from an A node to a B node and vice versa (including chained trips).

From the 100 individuals included in our sample, we identified a total of 1746 trips at the time of the data collection. These 100 people completed an average of 2.67 trips per day, which was within the range of previous similar statistics collected in Da Nang. In Kutani et al. (2015), they reported statistics on trip rate from the Da Nang residents’ survey conducted in 2008. These data showed that, excluding walking, individuals completed 2.3 trips per day, which was up to 2.9 trips per day when walking was included. In more recent report, the figure for trip rate in 2018 of Da Nang was reported at 2.98 trips/day (An et al. 2021). Trip rate of Da Nang is relatively high, this figure is almost equal to the figure of two other big cities of Vietnam, HCMC and Hanoi. The trip rates of these two cities are 3.0 trips/day (in 2018), and 3.15 trips/day (in 2017) respectively.

In terms of distance travelled, individuals in the sample covered 13.15 km per day on average, and around 4.96 km per trip. Furthermore, when chained trips (tours) were taken into account, we calculated that the average distance traveled by a person per tour was approximately 10.55 km, with 1.27 tours a day. Compared with developed countries such as the UK in Neves and Brand (2019), distance traveled per day was equivalent to 37.82 km (23.5 miles), or 24.67 km higher than that of Da Nang. This showed that, overall, individuals in our sample traveled within a relatively small radius within the city of Da Nang. The difference in daily average trip lengths between our calculation for Vietnam and studies conducted in other countries is attributed to the difference in habits of choosing a place to live and work. In Vietnam, the citizens here are more likely to consider the advantageous locational attributes when choosing their home/accomodation or workplace. In particular, they give priority to the accessibility and proximity to major public facilities and places, such as CBD areas, schools, shopping malls, main roads, markets and food vendors (Seo 2018; Won and Kim 2017). Therefore, people do not need to travel too far to access essential services, or to go to workplace. This may explain the difference in daily travel distance (24.67 km) between our study in Vietnam and other studies.

As shown in Table 2, 66.3% of all trips (n = 1159) recorded were less than 3 miles in length, of which 594 trips were shorter than 1 mile, accounting for 34%; and 565 trips were between 1–3 miles in length, accouting for 32%. Our finding is the first calculation of its kind in the context of Vietnam. Compared to previous research, percentage of short trip (< 3 miles) in our finding (66.3%) is relatively higher than that of Neves & Brand (2019), with 58.8% of recorded trips in the context of developed countries like UK. The main reasons of this difference were related to Vietnamese living habits, priorities when choosing accomodation or workplace, convenience and services accessibility as mentioned above (Seo 2018; Won and Kim 2017). This travel pattern is considered as a potential feature that helps to promote active travel modes as a feasible solution for urban transport of Da Nang. It will be discussed in details in the next parts.

**Table 2:** Key statistics on travel activity

Category (unit)	Mean	Std. Dev	Min	Max
Average distance traveled by trip (km/trip)	4.96	4.01	0.38	24.57
Average distance traveled by tour (km/tour)	10.55	9.65	0.9	73.7
Average distance traveled by day (km/day)	13.15	13.09	0.9	73.7
Trip rate (number of trip/person/day)	2.67	1.04	0.5	6.5
Tour rate (number of tour/person/day)	1.27	0.47	0.25	3

### GHG emissions from motorized transport

The GHG emissions (CO_2_e) were computed as the emission factors multiplied by trip distance. This resulted in a total of GHG emissions produced by the 100 individuals in our sample. On average, each person in our sample contributed weekly an average of about 9.19 kgCO_2_e, of which 7.62 kgCO_2_e is direct GHG emissions and 1.57 kgCO_2_e is indirect GHG emissions. As explained in calculation guideline (DBEIS & DEFRA 2020; DECC & DEFRA 2012), direct GHG emissions are attributable to the generation of CO_2_, CH_4_, N_2_O from burning fuels to drive vehicles, it indicates the “tailpipe emissions”. Meanwhile, an indirect emissions factor includes the GHG emissions that results from the extraction, transport, refining, purification or conversion of primary fuels. This means that we can see the emissions not just at the point of use by direct end-users, but also the emissions from every element of producing, distribution process of these fuels, in other words, its life-cycle emissions Table 3.

**Table 3:** Distribution of trips and tours by length

Length	Trip (n = 1746)		Tour (n = 829)	
	Freq	Percent (%)	Freq	Percent (%)
< 1.61 km (or < 1 mile)	594	34.02	127	15.32
1.61 – 4.83 km (or 1–3 miles)	565	32.36	214	25.81
4.83 – 8.05 km (or 3–5 miles)	304	17.41	129	15.56
8.05 – 16.09 km (or 5–10 miles)	205	11.74	213	25.69
16.09 – 40.23 km (or 10–25 miles)	78	4.47	129	15.56
40.23 – 80.47 km (or 25–50 miles)	0	0.00	17	2.05

Our derived computation of weekly GHG emissions on transport sector of 9.19 kgCO2e/capita is the solely computational effort so far in Vietnam, a country with a two-wheels motorcycle being responsible for the large proportion of GHG emissions. This figure is low when compared to previous studies in the same field. For example, a study by Brand et al. (2013) conducted in three UK cities (Cadiff, Kenilworth and Southampton) estimated a derived sample average of which was 35.1 kgCO2e/capita/week, or in Neves and Brand (2019), this figure is at 28.6 kgCO2e/capita/week for Cadiff city alone Table 4.

**Table 4:** Total amount of greenhouse gas (kgCO_2_e) emissions from motorcycles

	CO_2_	CH_4_	N_2_O	Total direct GHG	Total indirect GHG	Grand total GHG
	(1)	(2)	(3)	(4) = (1) + (2) + (3)	(5)	(6) = (4) + (5)
Conversion factors (kgCO_2_e/km)	0.08086	0.00161	0.00030	0.08277	0.01710	0.10
Average GHG emissions per trip (kgCO_2_e/trip/person) (^a^)	0.40112	0.00799	0.00149	0.41060	0.08483	0.49
Daily GHG emissions (kgCO_2_e/day/person) (^b^)	1.06344	0.02117	0.00395	1.08856	0.22489	1.31
Weekly GHG emissions (kgCO_2_e/week/person)	7.44408	0.14819	0.02756	7.61993	1.57423	9.19

*Conversion factors were adopted from DBEIS & DEFRA* (2020) *for direct GHG (CO*_*2*_*, CH*_*4*_*, N*_*2*_*O) and DEEC & *DEFRA (2012)* for indirect GHG. The fact that we are unable to exclude some of them who may travel by bicycle or walking in our estimation will underestimate GHG emissions per trip and daily GHG emissions*

*Note: (*^*a*^*) This figure was calculated by using the average travel distance/trip in *Table 1*; (*^*b*^*) This figure was calculated by using the average travel distance/day in *Table 1

However, it should be noted that there is a huge difference in private vehicle ownership between developing countries (e.g. Vietnam) and developed countries (e.g. the UK). While GHG emissions in the transport sector in developed countries such as the UK reported Neves and Brand (2019) and Brand et al. (2013) was attributed to car travel. For Vietnam case, due to the dominance of travelling by two-wheels motorcycles, this can account for the difference between the above calculations.

To appraise the data about trip purposes, and GHG emission classified by travel purposes, we excluded 829 trips with the purpose of going home (to-home trips) from our computation. Then, a total of 917 trips were grouped into six main purposes. As can be seen in Table 5, nearly half of the trips (48%) were travels to work or receive education. In other words, commuting to workplace or educational institutions is the most GHG emitting purpose of travel in our observed sample. Other purposes included shopping and food-buying (15.8%), social and leisure (10.7%), personal businesses (9.5%), eating out (8%), and visiting relatives, family (7.7%). The last two purposes, which we suggested that should be considered as the two more separated purposes in the Vietnamese case, account together for 15.7% of total trips.

**Table 5:** Some key statistics on travel activity by purposes

	Education and work	Shopping and food buying	Social and leisure	Personal business	Eating out	Visit relative, family
Proportion of trips by purposes (%)	48.20	15.81	10.69	9.49	8.07	7.74
Average distance by purposes (km/trip)	9.51	3.29	8.59	13.17	13.69	14.88
Daily tour rate (tour/day)	0.78	0.43	0.32	0.42	0.30	0.31
Weekly tour rate (tour/week)	5.46	3.00	2.24	2.94	2.10	2.17

On average, the distance travelled for education and work purposes was 9.5 km/trip. They need to travel further for their personal business (13.2 km/trip), eating out (13.7 km/trip), and visit relatives and family (14.9 km/trip). As expected, they choose nearby locations to shop and buy food (3.3 km/trip) and for social and leisure activities (8.6 km/trip). On an average week, people dedicated 5.5 tours/week to education and work, 3 tours/week to shopping and buying groceries, another 3 tours/week to personal business, and 2 tours/week each to social and leisure activities, eating out, and visiting relatives and family Table 6.

**Table 6:** Key results of the mode shift potential

Category	Unit	Criteria A	Criteria B
		Only shift to biking or walking the tours that contain 2 trips (home-based trip chain) and the tours are up to 6 miles in length	Only shift to biking or walking the tours that contain 2 trips (home-based trip chain) and the tours are up to 6 miles in length. Plus exclude all short trips for shopping purposes
	Count (trips)	974	722
Short motorcycle trips with potential to be replaced by active travel	Percentage share out of 1159 short trips (%)	84	62.3
	Percentage share out of 1746 total trips (%)	55.8	41.4
Grand total GHG emissions if taking mitigation	kg CO_2_e/person/day	1.03	1.08
Grand total GHG reduction if taking mitigation compared to baseline (^a^)	Reduction in kg CO2e/person/day	0.29	0.23
	Total potential mitigation (%)	21.7	17.8

*Note: (*^*a*^*) baseline is 1.31345 kg CO*_*2*_*e/person/day or 9.193 kg CO*_*2*_*e/person/week*

### Short trip substitution

This section will focus on short trips and their purposes. From there, we assess the feasibility of reducing GHG emissions by replacing short trips with active travel modes. Taking the travel purposes and the potential of this substitution into account will enable our estimations to be more conservative.

Following the definition of Beckx et al. (2013), a “short trip” can be defined as a trip with a length between 3 and 5 miles (equal to from 4.83 to 8.05 km). In a more conservative manner and to be able to compare, we applied the definition proposed by Neves and Brand (2019) for this research, which specified all trips under 3 miles as short trips. Accordingly, we identified over 65% (n = 1159) out of total trips being shorter than 3 miles or 4.83 km in length (Table 2). In other words, short motorcycle trips were responsible for 65% of GHG emissions, given that we applied fixed conversion factors in calculating CO_2_e.

**Fig. 3:**
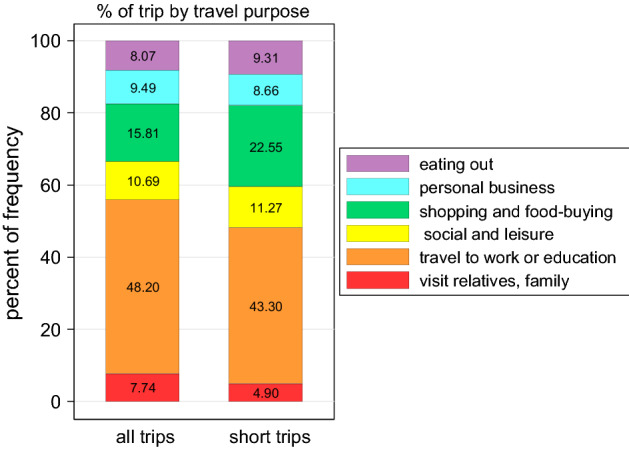
Proportion of trip purposes among all trips (N = 917) and short trips (N = 612). *Note: Number of trips here is different from total trips (n* = *1746) in *Table 2*, since we excluded 829 to-home trips*

Delving into the purposes of short trips, these short trips were mostly for work or education (43.3%). Other reasons to travel over short distances included shopping and food-buying (22.55%), or for social and leisure purposes (11.27%), personal businesses (8.66%), eating out (9.31%), and visiting relatives and family (4.90%). Among the short trips (compared to generally all trips) there were higher percentages for shopping and food-buying and eating out (Fig. 3). For these trips, people could be more flexible in choosing locations and tended to choose those close to their homes.

For most people, short trips were suitable for walking or biking (Frank et al. 2000; de Nazelle et al. 2010a; Pucher and Dijkstra 2003), so the 1,159 short motorcycle trips identified earlier could be moved into active transport modes. Apart from infrastructure and personal characteristics, however, trip attributes (e.g. trip purpose and its complexity) greatly affected the choice of travel mode (Beckx et al. 2013; de Nazelle et al. 2010a, b; Song et al. 2017). We thus evaluated the possibility of mode switching for these short-distance motorcycle trips by considering their purpose and complexity, such as whether short-distance trips were part of a chained trip.

Taking the realistic mode shift potential, switching a one-way short trip within chained trips or the long tour may not be realistic as people might not go back to the previous location and hence leave their bicycles behind. Visiting more than one location during a chained trip, though consisting of various manageable short trips in length altogether, may still make people feel reluctant to bike or walk as they may need to carry various things for different visiting locations/purposes. We hence only considered the tours up to 6 miles containing 2 short trips per tour (one-round trip). When shopping, passengers usually carry heavy items and groceries, so we then further filtered out all short trips with shopping purposes under the assumption that it would be unreasonable to expect a modal transition on these short trips.

Thus, among of 1,159 short trips that were considered in scope for substitution, the active travel modes could realistically replace 62% of short motorcycle trips (equivalent to 41% of all trips). Accordingly, we estimated that this mitigation option resulted in a reduction of 0.23 kg CO_2_e/person/day or nearly 18% reduction of GHG. If biking and walking were to replace all the shopping trips usually made on motorcycles, this would increase to 84% of short-distance trips (and reduce motorcycle-induced GHG emissions by 22%). Therefore, these results support the claim that walking and biking can play a critical role in promoting a low-GHG transportation system while having other positive effects on tackling traffic congestion, air pollution, and community health issues.

We compared our results with similar studies conducted in the UK (Beckx et al. 2013; Neves and Brand 2019) to provide a rich picture of travel behavior in developing countries versus developed countries. In reality, there should be more filtering trips and lower emissions due to escorting, being a passenger for some of the trips. However, there were no trips identified in such a situation in this profile. Nevertheless, the method of assessment was much more conservative in our study, for example, we assumed that tours could be shifted to 6 miles and could contain 2 trips only (visiting one location and returning home) whereas Neves and Brand (2019) suggested shifting the tours up to 8 miles instead 6 miles and the included short trips could be one-way trips. We also excluded all short trips to stores whereas Neves and Brand (2019) excluded only the trips to large retail stores and Beckx et al. (2013) included all trips. Still, our study revealed that cycling and walking could substitute for 41% of motorcycle trips whereas active travel could replace a fifth and only one-tenth of car-tracked trips in the studies by Neves and Brand (2019) and Beckx et al. (2013), respectively. Apart from the fact that our study had a higher percentage of trips recorded as being less than 3 miles (66% in our study compared to 59% in the UK in the study of Neves and Brand (2019)), this study revealed that people living relatively closer to their workplaces was one main reason behind those significant differences.

More people lived closely to their workplace in our study. About 77% of people who provided workplace information (n = 65), lived fewer than 5 miles from their workplace in our study site compared to 54% in the UK (Neves and Brand 2019). The main purpose of these short trips (43.3%) in our sample was for work or education. This is contrary to that in the UK where the main purpose of the short car trips was for shopping and food-buying (46.6%) whereas traveling to/from work or education totaled 19% only (Neves and Brand 2019). As a result, the possibility for mode shifting for short trips, though using similar (and stricter) criteria as research conducted in the UK, suggests a significantly greater potential for mode shifting. It also provides a good understanding of the more important role active travel can have in everyday travel in developing countries as compared to developed countries such as the UK.

## Conclusion

In this case study, by using reliable contact tracing data, we could innovatively identify the normal travel patterns of people in Da Nang city of Vietnam. Furthermore, our results found that active travel modes (i.e., walking or biking) have a remarkable potential, which can alternate short-distance motorcycle trips; and therefore, have a significant impact on the reduction of private vehicle-induced GHG emissions. Of all motorcycle trips, 65% are less than 3 miles. In fact, active travel modes can replace 62% of short motorcycle trips if considering travel patterns and constraints and reduce 18% of GHG emissions from motorized transport. For the calculation on 1746 trips in our sample, 41% of trips (and up to 56% when including shopping trips) had the potential to be replaced with walking or biking. These figures indicate that there is still room for incentive policy and infrastructure investment to support these active travel modes. From a comprehensive “co-benefit” standpoint, this transformation could act as a catalyst for addressing traffic congestion, air pollution, and even community health and well-being in urban areas.

Exploring more deeply into shopping trips, shopping accounted for nearly a quarter of the short trips in our sample, suggesting that we should see active travel as a modal choice worth considering for such activities. For example, equipping shopping vehicles to carry heavy goods (e.g. carts, trolleys, freight bikes), similar to the conclusions of Neves and Brand (2019), some of these journeys can be done entirely by bike or on foot. These simple actions could realistically contribute to the mitigation of a further 3% of GHG emissions from motorized travel.

We also acknowledge that we could not strongly infer possible shifting from using a motorcycle to walking/cycling simply based on the short trip distance by a motorcycle, especially when there is no intervention at all in the meantime. Because trip distances are short does not necessarily imply that people would shift to active transportation, and hence the anticipated GHG emissions might not be realistic. However, this first piece of empirical evidence on the potential of behavior changes on total emissions and therefore it could make the identification of the appropriate policies more reliable task, given that policies can be costly to implement.

Althought motorcycles are the dominant mode of transportation in Vietnam as mentioned above, car ownership is significantly increased due to the expansion of a fast-growing middle class, and trade liberalisation. This situation is similar to other developing countries such as Indonesia (Shigetomi et al. 2020). This may imply that the modal shift of developing countries toward automobile modes, to an extent parallel to what many developed countries’ feature, would be disastrous for global climate change, energy consumption, and GHG emissions long term. Under that scenario, it would be an impactful display of the consequences of not addressing transportation sustainability not just in Da Nang or Vietnam but also other under-studied developing countries.

## Data Availability

Not applicable.

## Footnotes

1 This is the name of a public dashboard operated by the Vietnamese Ministry of Health during the time of COVID-19 outbreaks. This is not an abbreviated name.

2 *The data we collected included information up to 27 July 2020, which was our cut-off time, implying that individual diaries varied in terms of the time range they covered.*
